# The Nitric Oxide System in Peripheral Artery Disease: Connection with Oxidative Stress and Biopterins

**DOI:** 10.3390/antiox9070590

**Published:** 2020-07-06

**Authors:** Ahmed Ismaeel, Evlampia Papoutsi, Dimitrios Miserlis, Ramon Lavado, Gleb Haynatzki, George P. Casale, William T. Bohannon, Robert S. Smith, Jack Leigh Eidson, Robert Brumberg, Aaron Hayson, Jeffrey S. Kirk, Carlos Castro, Ian Sawicki, Charalambos Konstantinou, Luke P. Brewster, Iraklis I. Pipinos, Panagiotis Koutakis

**Affiliations:** 1 Department of Nutrition, Food and Exercise Sciences, Florida State University, Tallahassee, FL 32304, USA; ai18@my.fsu.edu (A.I.); epapoutsi@fsu.edu (E.P.); 2 Department of Surgery, University of Texas Health Science Center San Antonio, San Antonio, TX 78229, USA; miserlisd@uthscsa.edu; 3 Department of Environmental Science, Baylor University, Waco, TX 76798, USA; ramon_lavado@baylor.edu; 4 Department of Biostatistics, University of Nebraska Medical Center, Omaha, NE 68198, USA; ghaynatzki@unmc.edu; 5 Department of Surgery, University of Nebraska Medical Center, Omaha, NE 68198, USA; gpcasale@unmc.edu (G.P.C.); ipipinos@unmc.edu (I.I.P.); 6 Department of Surgery, Baylor Scott & White Medical Center, Temple, TX 76508, USA; William.Bohannon@BSWHealth.org (W.T.B.); Robert.Smith@BSWHealth.org (R.S.S.); Jack.Eidson@BSWHealth.org (J.L.E.); Ian.Sawicki@BSWHealth.org (I.S.); 7 Vascular Surgery Associates, Tallahassee, FL 32308, USA; rbrumberg@vsafl.com (R.B.); awh2830@gmail.com (A.H.); 8 Department of Vascular Surgery, Capital Regional Medical Center, Tallahassee, FL 32308, USA; Jeffrey.kirk@hcahealthcare.com (J.S.K.); Carlos.Castro@hcahealthcare.com (C.C.); 9 Department of Electrical & Computer Engineering, Florida State University, Tallahassee, FL 32310, USA; konstantinou@caps.fsu.edu; 10 Department of Surgery, Emory University School of Medicine, Atlanta, GA 30322, USA; lbrewst@emory.edu; 11 Department of Surgery and Research Service, Veterans Affairs-Western Iowa Medical Center, Omaha, NE 68105, USA

**Keywords:** tetrahydrobiopterin, dihydrobiopterin, endothelial dysfunction

## Abstract

Peripheral artery disease (PAD) pathophysiology extends beyond hemodynamics to include other operating mechanisms, including endothelial dysfunction. Oxidative stress may be linked to endothelial dysfunction by reducing nitric oxide (NO) bioavailability. We aimed to investigate whether the NO system and its regulators are altered in the setting of PAD and to assess the relationship between NO bioavailability and oxidative stress. Sera from 35 patients with intermittent claudication (IC), 26 patients with critical limb ischemia (CLI), and 35 non-PAD controls were analyzed to determine levels of tetrahydrobiopterin (BH4), dihydrobiopterin (BH2), nitrate/nitrite (nitric oxides, or NOx), arginine, citrulline, asymmetric dimethylarginine (ADMA), symmetric dimethylarginine (SDMA), and the oxidative stress markers 8-Oxo-2′-deoxyguanosine (8-OHdG), 4-hydroxynonenal (4-HNE), advanced glycation end products (AGEs), and protein carbonyls. NOx was significantly lower in IC and CLI patients compared to controls in association with elevated oxidative stress, with the greatest NOx reductions observed in CLI. Compared with controls, IC and CLI patients had reduced BH4, elevated BH2, and a reduced BH4/BH2 ratio. SDMA, the arginine/SDMA ratio, and the arginine/ADMA ratio were significantly higher in CLI patients. The NO system and its regulators are significantly compromised in PAD. This dysregulation appears to be driven by increased oxidative stress and worsens as the disease progresses from claudication to CLI.

## 1. Introduction

Peripheral artery disease (PAD) is a vascular condition characterized by the narrowing and occlusion of the arteries supplying the lower extremities [1]. Symptomatic PAD patients present with intermittent claudication (IC), which is the milder and most common presentation of PAD, characterized by walking-induced leg pain that is relieved by rest, or with critical limb ischemia (CLI), the more severe and less common presentation of PAD, characterized by ischemic foot pain at rest and non-healing ulcers/gangrene [2].

The manifestations of PAD are the products of reduced blood flow (producing ischemia and ischemia/reperfusion) working in combination with a number of associated pathophysiologic mechanisms. The two best studied mechanisms operating in PAD include abnormal skeletal muscle metabolism and histology, better known as the myopathy of PAD, and endothelial dysfunction [3,4]. Endothelial dysfunction in PAD represents a decreased bioavailability of nitric oxide (NO) and is most commonly documented as impaired flow-mediated dilation (FMD) [5]. In patients with PAD, FMD that is closer to normal has been associated with improved exercise performance [6], while low FMD has been shown to independently predict cardiovascular risk and risk of leg amputation [7,8]. Oxidative stress is recognized as one of the major inducers of endothelial dysfunction [9] primarily working by decreasing NO bioactivity. In PAD, chronic, effort-induced cycles of ischemia-reperfusion (I/R) have been shown to lead to increased production of reactive oxygen species (ROS) and oxidative damage that contribute to the pathophysiology of the disease [10]. One key mechanism by which ROS affects NO generation is by reducing the levels of tetrahydrobiopterin (BH4). BH4 plays a critical role as a co-factor in the enzymatic activity of all nitric oxide synthase (NOS) enzymes, which produce NO from the biological precursor L-arginine [11]. Specifically, the binding of BH4 to NOS causes a conformational change that increases the binding affinity of L-arginine to NOS [12]. In conditions of elevated oxidative stress, BH4 can be oxidized to dihydrobiopterin (BH2) [13], which lacks co-factor activity and competes with BH4 for NOS binding. Furthermore, BH2 binding to eNOS can result in the “uncoupling” of L-arginine oxidation from electron transfer, which results in the formation of O_2_^−^ [14]. This process has been implicated in several disease states which are characterized by oxidative stress and endothelial dysfunction, including coronary artery disease and hypertension [15]. Thus, the maintenance of a balanced ratio of BH4/BH2 is thought to play a significant role in vascular health, and a reduced ratio of BH4/BH2 has been recognized as a marker of endothelial dysfunction and a critical determinant of eNOS uncoupling [16]. Additionally, recent work from our group has shown that the ratio of phenylalanine (Phe)/tyrosine (Tyr) is elevated in the serum of PAD patients compared to non-PAD controls and that the ratio is also increased with advancing disease stage [17]. This finding supports the presence of a significant BH4/BH2 imbalance in PAD, since BH4 is also a co-factor for phenylalanine hydroxylase, which catalyzes the conversion of Phe to Tyr, and the loss of BH4 by oxidation is believed to be a cause of elevated Phe/Tyr ratio [18].

In the present work, we hypothesized that the NO system and its regulators are significantly compromised in PAD, and we tested this by comparing the serum levels of the molecules that are involved in the bioavailability of NO in non-PAD controls (control) and symptomatic PAD patients presenting with either IC or CLI. We measured the serum levels of nitrate/nitrite (NOx, the final metabolites of NO commonly used to quantify NO bioavailability), BH4, BH2, arginine, citrulline, asymmetric dimethylarginine (ADMA), and symmetric dimethylarginine (SDMA). ADMA has been shown to act as an endogenous inhibitor of NOS by interfering with L-arginine [19], while SDMA has been shown to affect NO levels by potentially blocking cellular L-arginine uptake [20]. Finally, we also assessed the serum levels of protein carbonyls, 8-Oxo-2′-deoxyguanosine (8-OHdG), and 4-hydroxynonenal (4-HNE) in order to assess the relationship between NO bioavailability and oxidative stress.

## 2. Materials and Methods

### 2.1. Study Approval and Participants

Vascular surgeons at the University of Nebraska Medical Center (UNMC, 00707), Baylor Scott and White Hospital (BSWI, 160390), Vascular Surgery Associates Clinic (FSU 00272), and Capital Regional Medical Center (CRMC 0054) recruited 35 IC patients, 26 CLI patients, and 35 non-PAD controls under approved IRB protocols. This study complies with the Declaration of Helsinki, and informed consent was obtained from all participants. The diagnosis of IC or CLI was made following a physical and medical history examination, measurement of the ankle brachial index (ABI), and arteriography. All controls had normal blood flow to their extremities and were undergoing operations for conditions not related to PAD. These patients also had no history of PAD symptoms and normal ABIs at rest and after stress. They were all sex-matched and led sedentary lifestyles.

### 2.2. Sample Collection and Preparation

Thirty mL of blood was obtained from each patient and control after an overnight fast. Blood was immediately centrifuged (2000 *g*, 10 min, 4 °C), and serum was aliquoted into separate polypropylene tubes and immediately stored at −80 °C until time of analysis.

### 2.3. BH4 and BH2 Determination

Commercial BH4 and BH2 enzyme-linked immunosorbent assay (ELISA) microwell strip plate kits were used for the determination of BH4 and BH2 concentrations in the serum samples (Novus Biologicals, Centennial, CO, USA). This technique of detection uses BH4 antibody-BH4 antigen immunoabsorbency interactions and a horseradish peroxidase (HRP) colorimetric detection system. The BH4 ELISA kit showed an intra-assay precision of 5.7% coefficient of variation (CV) and an inter-assay precision CV of 5.4%. Similarly, a commercial BH2 ELISA kit was used for the determination of BH2 concentrations in the serum samples (Novus Biologicals), with an intra-assay precision of 5.6% and an inter-assay precision of 5.1%. Serum samples were thawed to room temperature prior to use and diluted two-fold prior to testing. All samples, controls, and standards were assayed in duplicate. The optical density of the wells was determined using an ELx808 absorbance microplate reader set to 450 nm (BioTek, Winooski, VT, USA).

### 2.4. Metabolite Determination

The concentrations of serum arginine, citrulline, ADMA, and SDMA were determined using the Biocrates AbsoluteIDQ p400 HR kit (Biocrates Life Science AG, Innsbruck, Austria), as previously described [17]. Briefly, the metabolites were analyzed on a Thermo Scientific UltiMate 3000 Rapid Separation Quaternary high performance liquid chromatography (HPLC) System (Thermo Scientific, Madison, WI, USA), connected to a QExactiveTM Focus Hybrid Quadrupole-OrbitrapTM Mass Spectrometer (Thermo Scientific), by liquid chromatography-tandem mass spectrometry (LC-MS/MS), after derivatization with phenylisothiocyanate, and they were quantified using internal standards and multiple reaction monitoring.

### 2.5. NOx Determination

We used a commercial Nitric Oxide Assay Kit (Invitrogen, Carlsbad, CA, USA) to determine serum NOx by the measurement of nitrate and nitrite, which are the final metabolites of NO. First, levels of endogenous nitrite were detected as products of the Griess reaction, with a colored azo dye that absorbs at 540 nm. Next, all the nitrate in the samples was converted into nitrite using the enzyme nitrate reductase, and the total nitrite was measured. To obtain the nitrate concentration in the samples, the endogenous nitrate was subtracted from the total nitrite value. Serum was diluted 1:2 for the first assay and 1:20 for the second assay. The intra-assay precision for nitrite was 2.4% CV and 1.5% CV for nitrate. The inter-assay precision was 2.9% CV for nitrite and 3.4% CV for nitrate. All samples, controls, and standards were assayed in duplicate. The optical density of the wells was determined using an ELx808 absorbance microplate reader set to 540 nm (BioTek, Winooski, VT, USA). Quantification of both nitrate and nitrite (referred to as nitric oxides or, simply, NOx) is believed to be an accurate method to determine total NO production [21].

### 2.6. 8-OHdG Assay

A commercial 8-OHdG ELISA kit (Abcam, Cambridge, UK) was used for the competitive quantitative measurement of 8-OH-dG in serum samples. The kit utilizes an 8-OH-dG-coated plate and an HRP-conjugated antibody. It is known that 8-OHdG is produced when DNA is oxidatively damaged by ROS or reactive nitrogen species (RNS), and it is a widely used biomarker for oxidative stress [22]. The intra-assay CV was determined as 5%. Samples were thawed to room temperature prior to use and diluted 1:20 in sample diluent. All samples, controls, and standards were assayed in duplicate. The optical density of the wells was determined using an ELx808 absorbance microplate reader set to 450 nm (BioTek, Winooski, VT, USA).

### 2.7. 4-HNE Adducts Assay

A commercial 4-HNE adduct competitive ELISA kit (Abcam, Cambridge, UK) was used for the quantification of 4-HNE adducts. It is known that 4-HNE is a byproduct of lipid peroxidation that is increased under conditions of oxidative stress [22], and 4-HNE is capable of reacting with amino acid residues in protein and forming stable adducts. Thus, the quantity of 4-HNE adduct in protein samples can be determined by comparing its absorbance to a known 4-HNE-BSA standard curve using an anti-HNE antibody and HRP conjugated secondary antibody. The intra-assay CV was determined as 7.5%. Serum samples were thawed to room temperature prior to use and diluted two-fold prior to testing. All samples, controls, and standards were assayed in duplicate. The optical density of the wells was determined using an ELx808 absorbance microplate reader set to 450 nm (BioTek, Winooski, VT, USA).

### 2.8. Protein Carbonyl Assay

A commercial protein carbonyl ELISA kit (Enzo Biochem, Inc., Farmingdale, NY, USA) was used for the measurement of protein carbonyls in the serum samples. Protein carbonyls are formed via oxidative mechanisms and are an established and sensitive index of oxidative stress [22]. This kit determines protein carbonyl quantity by derivatizing samples with dinitrophenylhydrazine (DNP) and probing the bound DNP with biotinylated anti-DNP antibody and streptavidin-linked HRP. A standard curve was prepared using serum albumin standards containing increasing concentrations of hypochlorous acid-oxidized protein. The intra-assay variation was 5% CV. Serum samples were thawed to room temperature prior to use. All samples, controls, and standards were assayed in duplicate. The optical density of the wells was determined using an ELx808 absorbance microplate reader set to 450 nm (BioTek, Winooski, VT, USA).

### 2.9. Advanced Glycation End Products Assay

Since diabetes is one of the major risk factors for PAD, we measured the levels of advanced glycation end products (AGEs), which are compounds formed by the glycation of macromolecules by reducing carbohydrates [23]. In diabetic patients, hyperglycemic conditions can allow glucose to form adducts with proteins, forming advanced glycation end products, which are thought to play a role in diabetic complications [23]. A commercial Oxiselect^TM^ Advanced Glycation End Product Competitive ELISA Kit (Cell Biolabs, San Diego, CA, USA) was used to measure the AGEs in the serum samples. This assay uses an AGE conjugate-coated plate, an anti-AGE antibody, and an HRP conjugated secondary antibody. The content of AGE adducts in the samples was determined from an AGE-bovine serum albumin (BSA) standard curve. Serum samples were thawed to room temperature prior to use and diluted two-fold prior to testing. All samples, controls, and standards were assayed in duplicate. The intra-assay variation was 4% CV. The optical density of the wells was determined using an ELx808 absorbance microplate reader set to 450 nm (BioTek, Winooski, VT, USA).

### 2.10. Statistical Analysis

Patient demographics and clinical characteristics (age, sex, risk factor prevalence, and ABI) between the IC, CLI, and control participants were compared using chi-square and Fisher exact tests for categorical variables and analysis of variance (ANOVA) for continuous variables. A one-way analysis of covariance (ANCOVA) was used to test differences in BH4 and BH2 concentrations as well as the ratio of BH4/BH2 between the IC, CLI, and control groups, controlling for significant covariates. A Pearson correlation was also calculated to test the association between ABI and BH4, BH2, and the BH4/BH2 ratio.

A one-way ANCOVA was also used to test differences in arginine, citrulline, ADMA, SDMA, the arginine/ADMA ratio, the arginine/SDMA ratio, NOx, protein carbonyls, and 4-HNE between groups. A Pearson correlation was further calculated to test the association between the metabolites and ratios, NOx, protein carbonylation, 8-OHdG, and 4-HNE with ABI, as well as BH4, BH2, and the BH4/BH2 ratio. An independent sample t-test was used to test the difference in AGEs between diabetic and non-diabetic patients. A Pearson correlation was also calculated to test the association between AGEs and ABI as well as NOx. All analyses were performed using SPSS statistical software version 25 (IBM, Armonk, NY, USA). Significance was set at α < 0.05.

## 3. Results

### 3.1. Patient Demographics

The participant demographics and clinical characteristics are presented in Table 1. Both IC and CLI patients had lower ABI values than the controls (control: 1.08 ± 0.05, IC: 0.55 ± 0.25, CLI: 0.27 ± 0.28, *p* < 0.001). IC and CLI patients both had higher rates of diabetes mellitus (DM) than the controls (*p* = 0.008). No other differences between groups were observed.

### 3.2. BH4 Concentrations

**Figure 1:**
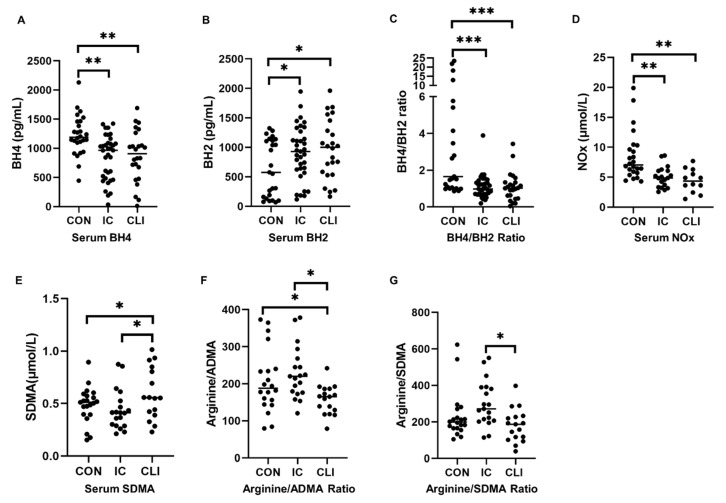
Levels of nitric oxides (NOx) and regulators of nitric oxide (NO) synthesis. The concentrations of these compounds were measured in the sera of patients with intermittent claudication (IC), critical limb ischemia (CLI), and non-peripheral artery disease (PAD) controls. (**A**), tetrahydriopterin (BH4) concentrations; (**B**), dihydrobiopterin (BH2) concentrations; (**C**), the ratio of BH4/BH2; (**D**), NOx concentrations; (**E**), symmetric dimethylarginine (SDMA) concentrations; (**F**), the ratio of arginine/asymmetric dimethylarginine (ADMA); (**G**), the ratio of arginine/SDMA. Bars indicate median, and *p* values were calculated by analysis of covariance (ANCOVA) test. * represents *p* < 0.05, ** represents *p* < 0.01, *** represents *p* < 0.0001.

BH4 levels were significantly lower in IC and CLI patients compared to controls (Table A1) (control: 1226.16 ± 342.39 pg/mL, IC: 846.01 ± 384.91 pg/mL, CLI: 870.04 ± 445.29 pg/mL, *p* = 0.001) (Figure 1A). Additionally, there was a significant association between BH4 concentrations and ABI (r = 0.336, *p* = 0.002).

### 3.3. BH2 Concentrations

BH2 levels were significantly elevated in IC and CLI patients compared to controls (control: 646.87 ± 478.70 pg/mL, IC: 907.19 ± 459.56 pg/mL, CLI: 969.16 ± 508.59 pg/mL, *p* = 0.045) (Figure 1B). There was also a significant association between BH2 concentrations and ABI (r = −0.221, *p* = 0.05).

### 3.4. BH4/BH2 Ratio

The ratio of BH4/BH2 was significantly lower in IC and CLI patients compared to controls (control: 5.28 ± 6.71, IC: 1.08 ± 0.64, CLI: 1.09 ± 0.78, *p* < 0.001) (Figure 1C). The BH4/BH2 ratio was significantly associated with the ABI (r = 0.381, *p* = 0.001).

### 3.5. NOx

Both IC and CLI patients demonstrated reduced serum NOx levels compared to controls (control: 8.49 ± 4.08 μmol/L, IC: 5.01 ± 1.75 μmol/L, CLI: 4.40 ± 1.95 μmol/L, *p* < 0.0001) (Figure 1D). Additionally, there was a significant positive correlation between NOx and the ABI (r = 0.403, *p* = 0.002). Specifically, endogenous nitrite concentrations were significantly lower in CLI patients (control: 3.89 ± 3.23 μmol/L, IC: 3.57 ± 2.32 μmol/L, CLI: 1.54 ± 1.08 μmol/L, *p* = 0.037), and nitrate levels were lower in both IC and CLI patients (control: 4.56 ± 3.01 μmol/L, IC: 1.72 ± 1.43 μmol/L, CLI: 2.42 ± 1.59 μmol/L, *p* < 0.0001).

### 3.6. Metabolites

SDMA concentrations were significantly higher in CLI patients than in both IC patients and controls (control: 0.49 ± 0.17 μmol/L, IC: 0.44 ± 0.19 μmol/L, CLI: 0.66 ± 0.35 μmol/L, *p* = 0.026) (Table A1) (Figure 1E). The arginine/ADMA ratio was significantly lower in CLI patients compared to both IC patients and controls (control: 206.82 ± 86.10, IC: 229.23 ± 70.56, CLI: 157.05 ± 38.94, *p* = 0.01) (Figure 1F). The arginine/SDMA ratio was significantly lower in CLI patients compared to IC patients (control: 257.32 ± 168.51, IC: 300.34 ± 124.14, CLI: 180.38 ± 90.93, *p* = 0.033) (Figure 1G). There were no differences between groups for arginine (*p* = 0.106), citrulline (*p* = 0.461), or ADMA (*p* = 0.134).

There was a significant association between arginine and the BH4/BH2 ratio (r = 0.373, *p* = 0.004) and SDMA and the ABI (r = −0.285, *p* = 0.032). Within the PAD subset (IC and CLI), the ABI was significantly correlated with ADMA (r = −0.349, *p* = 0.037), SDMA (r = −0.407, *p* = 0.014), the arginine/ADMA ratio (r = 0.438, *p* = 0.008), and the arginine/SDMA ratio (r = 0.374, *p* = 0.025).

### 3.7. 8-OHdG

**Figure 2:**
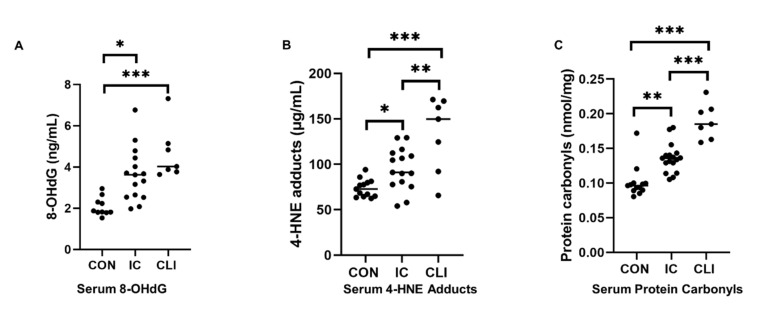
Levels of oxidative stress markers. Levels of markers of DNA, lipid, and protein oxidation were measured in the sera of patients with intermittent claudication (IC), critical limb ischemia (CLI), and non-PAD controls. (**A**), levels of 8-Oxo-2′-deoxyguanosine (8-OHdG); (**B**), levels of 4-hydroxynonenal (4-HNE); (**C**), levels of protein carbonyls. Bars indicate median, and *p* values were calculated by ANCOVA test. * represents *p* < 0.05, ** represents *p* < 0.01, *** represents *p* < 0.0001.

**Figure 3:**
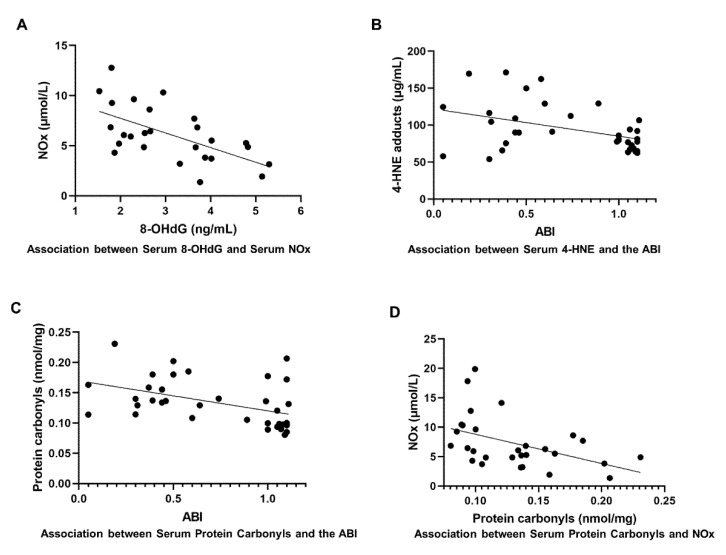
Associations between oxidative stress markers with nitric oxides (NOx) and ankle-brachial index (ABI). A Pearson correlation was calculated to test the association between variables. (**A**), There was a significant association between 8-OHdG and NOx levels (r = −0.602, *p* = 0.001); (**B**), 4-hydroxynonenal (4-HNE) levels and the ABI (r = −0.409, *p* = 0.015); (**C**), protein carbonyl levels and the ABI (r = −0.439, *p* = 0.007); and (**D**), protein carbonyl and NOx levels (r = −0.457, *p* = 0.013).

Serum concentrations of 8-OHdG were significantly higher in both IC and CLI patients compared to controls (control: 2.53 ± 1.07 ng/mL, IC: 3.63 ± 1.29 ng/mL, CLI: 4.65 ± 1.30 ng/mL, *p* = 0.002) (Table A1) (Figure 2A). There was a moderate significant negative association between 8-OHdG and NOx levels as well (r = −0.602, *p* = 0.001) (Figure 3A).

### 3.8. 4-HNE Adducts

Serum levels of 4-HNE adducts were significantly higher in IC and CLI patients compared to controls (control: 73.79 ± 9.81 μg/mL, IC: 94.96 ± 23.19, CLI: 133.64 ± 41.27, *p* < 0.0001) (Figure 2B). The 4-HNE adduct levels were also negatively associated with the ABI (r = −0.409, *p* = 0.015) (Figure 3B).

### 3.9. Protein Carbonyls

The quantity of serum protein carbonyls was significantly higher in both IC and CLI patients compared to controls (control: 0.10 ± 0.02 nmol/mg, IC: 0.13 ± 0.04 nmol/mg, CLI: 0.19 ± 0.03, *p* < 0.0001) (Figure 2C). There was a significant inverse correlation between protein carbonyl levels and both the ABI (r = −0.439, *p* = 0.007) (Figure 3C) and the NOx levels (r = −0.457, *p* = 0.013) (Figure 3D).

### 3.10. AGEs

**Figure 4:**
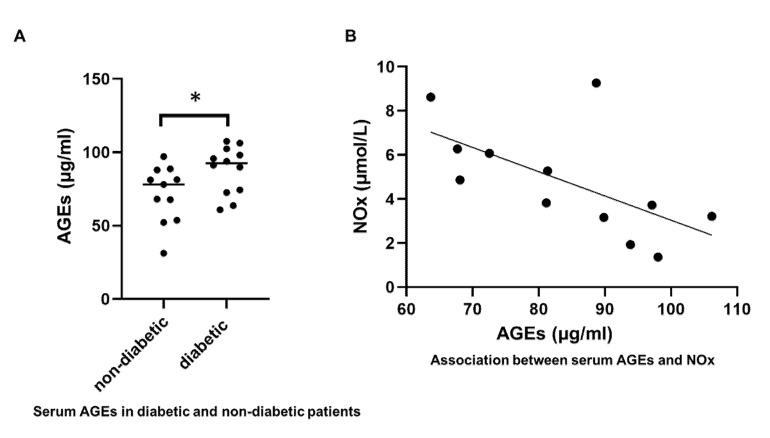
Serum advanced glycation end product (AGE) levels. (**A**), an independent sample t-test was used to test the difference between AGE levels in non-diabetic and diabetic patients. There was a significant difference in serum AGEs between non-diabetic and diabetic patients (*p* = 0.039). Bars indicate median, * represents *p* < 0.05. (**B**), A Pearson correlation was calculated to test the association between serum AGEs and serum NOx. There was a significant association between AGE levels and NOx (r = −0.62, *p* = 0.03).

The level of serum AGEs was significantly higher in PAD (both IC and CLI patients were included) diabetic patients (88.00 ± 16.18 μg/mL) compared to non-diabetic PAD patients (71.58 ± 19.48 μg/mL), *p* = 0.039 (Figure 4A). Serum AGEs were not significantly associated with the ABI (r = 0.30, *p* = 0.24). However, interestingly, there was a significant negative association between serum AGEs and serum NOx levels (r = −0.62, *p* = 0.03) (Figure 4B).

## 4. Discussion

In this study, we present data that support our hypothesis that the NO system and its regulators are significantly compromised in PAD. Firstly, our measurements demonstrate that serum NOx was significantly lower in both IC and CLI patients compared to non-PAD controls. This reduction in NO was associated with a compromised biopterin system, as levels of BH4 were significantly lower in both IC and CLI patients compared to controls, with no difference between IC and CLI patients. Furthermore, BH2 levels were significantly higher in IC and CLI patients compared to controls, and the BH4/BH2 ratio was lower in IC and CLI patients compared to controls, all of which point to the increased conversion of BH4 to BH2 by oxidation.

IC and CLI patients also demonstrated significantly greater oxidative stress compared to controls, as evidenced by their greater DNA, lipid, and protein oxidation. Oxidative stress is a well-studied aspect of PAD’s pathophysiology. During walking, patients experience lower extremity ischemia because the demands of the working muscles for increased blood flow cannot be met by the blocked arterial supply. Upon rest, blood flow demands return to the baseline and the patient’s legs experience reperfusion. This process of ischemia/reperfusion (I/R) is associated with increased ROS formation and leads to oxidative damage in the PAD legs [10]. One of the major sources of ROS in PAD is believed to be the mitochondria [24]. In PAD skeletal muscle, the activity and mitochondrial respiration of the electron transport chain complexes I, III, and IV are compromised, in association with elevated oxidative stress markers [25]. Likewise, PAD is also associated with compromised antioxidant defense systems. Specifically, manganese superoxide dismutase (MnSOD) [26], glutathione peroxidase [26], and scavenger antioxidants, including vitamin C, have also been shown to be reduced in PAD patients, in association with increasing disease severity [27]. Finally, circulating antioxidant capacity is also reduced in PAD patients and associated with decreasing walking performance [28]. Thus, increased ROS production, along with compromised antioxidant defense systems, has been shown to play an important role in the pathophysiology of PAD. In this study, serum levels of 8-OHdG, 4-HNE, and protein carbonyls were elevated in IC patients, with further elevations in the CLI patients. These findings are consistent with our previous work in gastrocnemius myofibers [29], as well as that of others on the serum of PAD patients [30]. We also found that increased oxidative stress, measured as levels of 8-OHdG as well as protein carbonyls, is associated with the decreased NOx levels in PAD, providing further evidence that increased ROS may play a role in driving the reduced NO bioavailability in these patients. Serum concentrations of SDMA were significantly higher in CLI patients, and the arginine/ADMA and arginine/SDMA ratio were significantly lower in CLI patients compared to IC patients and controls. Notably, the enzyme that produces ADMA, protein N-arginine methyltransferase (PRMT), is thought to be activated by oxidative stress [31], while the activity of the enzyme dimethylarginine dimethylaminohydrolase (DDAH), which degrades ADMA, is reduced by oxidative stress [32]. Several clinical studies have reported elevated ADMA and SDMA levels in diseases such as chronic kidney disease, type 2 diabetes, and cardiovascular disease, and in these diseases, levels of ADMA and SDMA have been found to correlate with negative clinical outcomes [33,34,35]. The ratio of arginine/ADMA and arginine/SDMA are also believed to be potential risk markers for vascular dysfunction [36]. Thus, it is possible that the increased oxidative stress in CLI patients, compared to both controls and IC patients, may be driving the increases in ADMA and SDMA.

Another finding of the present study was that, as expected, AGEs were elevated in the sera of diabetic PAD patients. Although the level of AGEs was not associated with the ABI, there was a significant negative association between AGEs and serum NOx levels. AGEs have been shown to contribute to chronic disease by inducing cellular dysfunction by the activation of cell surface receptors, referred to as receptors for AGEs (RAGE) [37]. AGEs can increase ROS production as well, and these molecules are believed to be a factor in DM-induced oxidative stress and tissue injury. Previously, diabetic PAD patients have been shown to exhibit elevated levels of AGEs in sera, in association with reduced antioxidant status [38]. The non-invasive measurement of AGEs using skin autofluorescence (SAF) has also demonstrated associations with all-cause mortality, major adverse cardiovascular events, and amputation in PAD patients [39,40,41,42]. Here, we show that AGEs may also be related to endothelial dysfunction in PAD via reduced NO bioavailability.

PAD patients, even those who are asymptomatic, suffer from walking impairments, substantial functional limitations, increased cardiovascular events, and poor quality of life, all of which worsen with advancing disease stage [43,44]. Numerous works from our group and others have established that, while PAD is initiated as the product of atherosclerotic blockages in the arterial supply of the legs, a number of key mechanisms are initiated and operate in association with the hemodynamic compromise in order to produce the significant symptoms, impairments and cardiovascular morbidity of PAD. One of the most recently described of such mechanisms is endothelial dysfunction, mainly a result of reduced production or bioavailability of NO, with accumulating data demonstrating that measures of endothelial dysfunction are associated with functional compromise and increased cardiovascular events in PAD patients [7,8]. Our data demonstrate that the NO system is significantly impaired in PAD, and this appears to be associated with increased oxidative stress, which may act by oxidation of BH4 and by alteration of the activities of the enzymes involved in the metabolism of SDMA and ADMA. BH4 is of particular interest in this process because it regulates NO and ROS production by NO synthase and is very vulnerable to depletion, thereby providing a potential mechanism contributing to the pathophysiology and manifestations of PAD and a potential target for therapeutic intervention.

Current options for PAD management are limited, and only two medications, cilostazol and pentoxifylline, are approved for claudication [2,45]. Regarding pentoxifylline, the overall benefit remains uncertain, as studies have had a large degree of variability in results [46]. While cilostazol seems more promising, the drug does not affect quality of life, mortality, or risk of cardiovascular events [47]. Instead, it seems to be modestly effective for improving walking distance in PAD patients [48]. Exercise training, especially supervised treadmill exercise, is recognized as a primary treatment option for PAD [43,49]. The main improvements reported as a result of exercise training in PAD are also pain-free walking distance, and, importantly, exercise training fails to restore hemodynamics [50,51]. Finally, in the case of CLI or disabling claudication, revascularization is recommended [52]. However, major limitations of these procedures include restenosis and thrombosis. In fact, patency rates of limb revascularizations are estimated to be as low as 56% at 1 year and 30% at 5 years [53,54]. Readmission rates due to complications can also be as high as 17% [53,54]. Thus, there is a great need for finding novel therapeutics and targets for intervention for PAD patients. Notably, BH4 supplementation has been previously tested and shown to improve endothelial function in smokers, as well as in patients with diabetes mellitus, hypocholesteremia, and coronary artery disease [55,56,57,58,59]. In addition to vasodilation, endothelial NOS (eNOS) and NO may also play a role in regulating the mobilization, function, and differentiation of endothelial progenitor cells, thereby affecting angiogenesis [60]. Furthermore, eNOS has been shown to be a mediator for in vivo angiogenesis in response to tissue ischemia [61] and, as a cofactor for eNOS, BH4 may also be important for these functions. Furthermore, BH4 has been shown, in vitro, to increase endothelial cell proliferation and tube formation in vascular endothelial cells from diabetic rats as well as bovine aorta [62]. Thus, the benefits of BH4 supplementation may also be related to their pro-angiogenic effects. Importantly, BH4 deficiency may also result in other complications, in addition to reduced NO bioavailability, since BH4 has other important functions in human biochemistry. For example, it is also a cofactor for tyrosine hydroxylase, which catalyzes the formation of L-DOPA, the dopamine precursor, from the amino acid tyrosine [63]. More recently, BH4 has also been shown to be important for T cell maturation and proliferation. In fact, mitochondrial respiration is reduced in T cells deficient in BH4 [64]. BH4 is also necessary for T-cell autoimmunity and inflammation. Interestingly, the overexpression of BH4 and treatment with BH4 were shown to promote anticancer immunity in a mouse model [64]. Treatment of a BH4 deficiency can be accomplished either by BH4 supplementation or with the use of antioxidants. Use of the mitochondrial-targeted antioxidant MitoTEMPO has been shown to increase the BH4/BH2 ratio in vitro [65]. In other studies in PAD patients, certain antioxidants targeting NADPH oxidase, a source of ROS, including propionyl-L-carnitine (PLC) and dark chocolate, have been shown to decrease serum markers of oxidative stress and increase serum NOx, the marker of NO biosynthesis [66,67]. Future studies should also assess whether vascular interventions (i.e., revascularization) improve NO bioavailability and the regulators of NO synthesis in PAD patients.

A limitation of this work is that we are not presenting functional data from the participants to address the extent to which the mechanisms we describe are related to the impaired limb function of PAD. Furthermore, this is a descriptive study and, therefore, its principal limitation is that it cannot establish cause and effect linkages among arterial blockages, NO bioavailability, oxidative stress, and BH4 depletion. Future studies should confirm and further analyze these mechanisms using in vitro studies. However, based on detailed human data, we demonstrate that the NO system and its regulators are significantly compromised in PAD and that this impairment is associated with increased oxidative stress and worsens as the disease progresses from claudication to CLI. High quality human data are needed for improving our knowledge of the mechanisms operating to produce PAD, for identifying the biomarkers of relevance to these underlying mechanisms, and, most importantly, for developing specific therapies for PAD patients. Several therapies, including the exogenous administration of BH4 and antioxidant medications, have been proposed for the management of endothelial dysfunction [15] and may be promising approaches for the management of PAD and its associated walking impairments and other local and systemic complications.

## 5. Conclusions

In conclusion, we provide evidence that NO bioavailability is reduced in PAD, which may be the result of enhanced oxidative stress and BH4 depletion. The recognition of the degree of endothelial dysfunction and its regulators in PAD may form the basis for better understanding PAD pathophysiology and for developing new therapeutic strategies in this disease. Our findings warrant further assessment of contributors such as NO, BH4, ADMA, SDMA, and ROS in a larger sample of PAD patients and in association with walking endurance, morbidity, and mortality. Since endothelial dysfunction is associated with mortality and cardiovascular events in PAD patients, this may implicate BH4/BH2 and NO as novel biomarkers that may be useful in the risk stratification of PAD patients, in the prediction of clinical outcomes, and as potential targets for PAD therapy.

## Figures and Tables

**Table 1:** Participant demographics at enrolment.

	Control (*n* = 35)	IC (*n* = 35)	CLI (*n* = 26)	*p*
Age (years)	62.11 ± 7.83	62.71 ± 8.93	64.54 ± 9.35	0.544
Male sex (%)	72.2%	86.8%	84.6%	0.466
ABI	1.08 ± 0.05	0.55 ± 0.25	0.27 ± 0.28	**<0.001**
**Risk factors (%)**				
Tobacco use				**0.005**
Current	24%	43.2%	50.0%	
Never	40%	5.7%	30.8%	
Former	36%	51.4%	19.2%	
Hypertension	68%	82.9%	88.5%	0.164
Diabetes mellitus	16%	34.3%	57.7%	**0.008**
Coronary artery disease	28%	37.1%	46.2%	0.407
Obesity	40.0%	20.0%	19.2%	0.146
Dyslipidemia	76.0%	68.6%	57.7%	0.372

Note: Participant demographics of control, intermittent claudication (IC), and critical limb ischemia (CLI) patients. Data presented as mean ± standard deviation. ABI represents ankle-brachial index. Values presented in the column “*p*” represent the overall difference between the three groups by chi-square and Fisher exact tests; bold font indicates significant difference between groups (*p* < 0.05).

## Appendix A

**Table A1:** Concentrations of nitric oxide bioavailability, nitric oxide synthesis regulators, and oxidative stress markers.

	Control (*n* = 26)	IC (*n* = 35)	CLI (*n* = 24)	*p*
**BH4 (pg/mL)**	1226.16 ± 342.39	846.01 ± 384.91 *	870.04 ± 445.29 *	**0.001**
**BH2 (pg/mL)**	646.87 ± 478.70	907.19 ± 459.56 *	969.16 ± 508.59 *	**0.045**
**BH4/BH2**	5.28 ± 6.71	1.08 ± 0.64 *	1.09 ± 0.78 *	**<0.001**
**N** **itrite (µmol/L)**	3.89 ± 3.23	3.57 ± 2.32	1.54 ± 1.08 *	**0.037**
**Nitrate (µmol/L)**	4.56 ± 3.01	1.72 ± 1.43 *	2.42 ± 1.59 *	**<0.0001**
**NOx (µmol/L)**	8.49 ± 4.08	5.01 ± 1.75 *	4.40 ± 1.95 *	**<0.0001**
**Arginine (µmol/L)**	107.84 ± 33.88	113.51 ± 21.77	94.28 ± 23.55	0.106
**Citrulline (µmol/L)**	31.98 ± 12.18	32.57 ± 14.08	38.47 ± 24.42	0.461
**ADMA (µmol/L)**	0.56 ± 0.17	0.52 ± 0.10	0.62 ± 0.15	0.134
**SDMA (µmol/L)**	0.49 ± 0.17	0.44 ± 0.19	0.66 ± 0.35 *^,†^	**0.026**
**Arginine/ADMA**	206.82 ± 86.1	229.23 ± 70.56	157.05 ± 38.94 *^,^^†^	**0.01**
**Arginine/SDMA**	257.32 ± 168.51	300.34 ± 124.14	180.38 ± 90.93 ^†^	**0.033**
**8-OHdG (ng/mL)**	2.53 ± 1.07	3.63 ± 1.29 *	4.65 ± 1.30 *	**0.002**
**4-HNE adducts (µg/mL)**	73.79 ± 9.81	94.96 ± 23.19 *	133.64 ± 41.27 *^,†^	**<0.0001**
**Protein carbonyls (nmol/mg)**	0.10 ± 0.02	0.13 ± 0.04 *	0.19 ± 0.03 *^,†^	**<0.0001**

Note: Data presented as mean ± standard deviation. The values presented in the column “*p*” represent the overall difference between three groups; bold font indicates a significant difference between groups (*p* < 0.05); *post-hoc* differences in comparisons between individual groups are denoted as: * = significant difference from control, *p* < 0.05, † = significant difference from IC, *p* < 0.05.
